# Association between sleep duration and the risk of hyperhomocysteinemia among adults in the United States: National Health and Nutrition Examination Survey, 2005–2006

**DOI:** 10.1007/s41105-024-00538-9

**Published:** 2024-06-22

**Authors:** Liang Xu, Yuehui Jia, Qiong Xiao

**Affiliations:** 1 Department of State-Owned Assets Administration, Qiqihar Medical University, Qiqihar, Heilongjiang Province 161000 People’s Republic of China; 2 Department of Epidemiology and Biostatistics, College of Public Health, Qiqihar Medical University, Qiqihar, Heilongjiang Province 161000 People’s Republic of China; 3 Department of Enrollment and Employment, Qiqihar Medical University, 333 Bukui North Street, Jianhua District, Qiqihar, Heilongjiang Province 161000 People’s Republic of China

**Keywords:** Sleep duration, Hyperhomocysteinemia, Logistic regression model, Restrictive cubic spline, National health nutrition examination survey

## Abstract

The study aimed to discuss the association between sleep duration and the risk of hyperhomocysteinemia (Hhcy). This cross-sectional study included 4173 adults (≥ 20 years) from the National Health and Nutrition Examination Survey 2005–2006. According to their sleep duration, participants were divided into five subgroups. Multivariate logistic regression analysis models and restrictive cubic spline regressions were used to explore the association between sleep duration and the risk of Hhcy. Compared with the participants who sleep 7 h, sleep deprivation (≤ 5 h) increased the risk of Hhcy, odds ratio (OR) 1.68 (95% confidence interval (CI) 1.06–2.68); Excessive sleep (≥ 9 h) also increased the risk of Hhcy, OR 1.86 (95% CI 1.09–3.14) after adjusting for a series of confounding factors in the entire population. The risk of Hhcy was distributed in a U-shape with sleep duration. Similar results were demonstrated in obese populations. The association between sleep duration and the risk of Hhcy is U-shaped. Both sleep deprivation and excessive sleep can increase the risk of Hhcy.

## Introduction

Sleep represents an essential element for health and well-being, including cognitive performance, physiologic processes, emotion regulation, physical development, and quality of life [1]. Sleep duration is the most frequently investigated sleep measure in relation to health. Insufficient sleep is well recognized and declared as a public health problem by the Centers for Disease Control and Prevention in the United States, and has a high impact on a country’s economy [2, 3]. However, long sleep duration is also positively associated with chronic diseases, such as obesity, type 2 diabetes, hypertension, and cardiovascular disease among adults [4, 5]. Sleep is closely related to circadian rhythms and sleep deficiencies associates with insomnia, sleep apnea, narcolepsy, and circadian misalignment [6]. In general, a sleep duration of 7–8 h per day is considered appropriate for optimal mental- and physical-health [7].

Homocysteine is a sulfur-containing amino acid that is synthesized by the liver and plays a role in the metabolism of methionine [8]. Homocysteinemia (Hcy) is the elevation of the homocysteine level in blood. Plasma total Hcy is a risk factor for cardiovascular disease, adverse pregnancy outcomes, and impaired cognitive function [9, 10]. In addition, there is evidence that plasma total Hcy may be associated with the development of schizophrenia, cognitive impairment or bipolar disorder [11, 12]. Previous studies have demonstrated that serum Hcy and body time are genetically interdependent [13]. Sleep is closely related to circadian rhythms. Melatonin, a hormone involved in circadian rhythm entrainment [14], has been shown to synchronize the circadian rhythms, and improves the onset, duration and quality of sleep. Previous studies have postulated that dysregulation of melatonin rhythms is the driving force behind sleep and circadian disorders, and causes hyperhomocysteinemia (Hhcy) because of disruption of homocysteine metabolism [15, 16]. One recent study investigated nutritional biomarkers and sleep conditions and found that short sleep duration might be associated with increased serum homocysteine levels [17]. Meanwhile, other studies have reported that insomnia or other sleep disorders is associated with serum homocysteine levels, and is an independent risk factor for Hhcy [17, 18].

However, the relationship between sleep duration and the risk of Hhcy has not been reported and the mechanism is unclear. Based on the above, we hypothesized that extreme sleep durations (≤ 5 h, ≥ 9 h) might be associated with increased serum homocysteine levels.

Therefore, the aim of this study was to investigate the relationship between sleep duration and Hhcy using data from the National Health and Nutrition Examination Survey (NHANES), and dietary factors served as important confounding variables that were modified during the study.

## Methods

### Study population

The NHANES is a population-based survey designed to collect information on the health and nutritional status of the household population in the United States and is jointly developed by the Centers for Disease Control and Prevention and the National Center for Health Statistics (NCHS). Detailed information on NHANES has previously been provided [19]. The survey data and questionnaire are available on the website to download (http://www.cdc.gov/nchs/nhanes.htm).

A total of 6139 adults were selected from the NHANES database from 2005 to 2006. Participants were excluded when the adults were pregnant females (*n* = 307) or had missing information on sleep duration and serum Hcy (*n* = 1659). Eventually, a total of 4137 subjects were analyzed in the present study.

### Assessment of covariates and outcome

Dietary factors include total energy (kcal/day), fat (g/day), dietary fiber (g/day), carbohydrate (g/day), protein (g/day), saturated fatty acids (SFA, g/day), monounsaturated fatty acids (MUFA, g/day), polyunsaturated fatty acids (PUFA, g/day), folic acid (mg/day), vitamin B12 (mg/day) intake and alcohol consumption (drinks/week). Nondietary factors include age (years), gender (men, women), race (Mexican American, Other Hispanic, Non-Hispanic White, Non-Hispanic Black, Other Race), smoking (never smoked, current smoker, ex-smoker), annual household income (≥ $20,000, < $20,000), body mass index (BMI, kg/m^2^), and hypertension (yes, no). Hypertension was defined as persistent systolic blood pressure measurements of ≥ 140 mm of mercury (mmHg) and/or diastolic blood pressure of 90 mmHg. BMI was calculated as weight in kilograms divided by the square of height in meters. Biochemical indicators: high-density lipoprotein cholesterol (HDL-C, mg/dL), serum total cholesterol (TC, mg/dL), and uric acid (UA, mg/dL). The main outcome is Hhcy which is diagnosed by serum Hcy > 12 μmol/L [20].

### Statistical analysis

All statistical analyses were performed using R 4.1.0 (www.r-project.org/). A two-sided *p* < 0.05 was considered statistically significant. Among the study variables, continuous variables were expressed as mean and standard deviation, and categorical variables were expressed as frequency (percentage, %). The distribution differences of the study variables were analyzed for different sleep-duration groups, and general linear models were used to analyze whether the continuous variables were statistically different among the groups. Chi-square tests were used to analyze the statistical differences in the categorical variables among the groups.

In this study, 4173 adults were selected to explore the relationship between sleep duration and the risk of Hhcy by integrating population data from the NHANES database during 2005–2006. According to sleep duration of the population, the sample was divided into five groups: group Q1 was the population with sleep duration of 7 h, group Q2 was the population with sleep duration of less than or equal to 5 h, group Q3 was the population with sleep duration of 6 h, group Q4 was the population with sleep duration of 8 h, and group Q5 was the population with sleep duration of more than or equal to 9 h.

A multivariate logistic regression model was used to explore the association between sleep duration and the risk of Hhcy, and the odds ratio (OR) value and 95% confidence interval (CI) were calculated. In the multivariate logistic regression model analysis, model 1 was adjusted for age, gender, smoking, drinking, annual household income, and race; model 2 was further adjusted for total energy, fat, dietary fiber, carbohydrate, protein, SFA, MUFA, PUFA, folic acid, and vitamin B12 based on model 1. Model 3 continued to adjust for HDL, TC, and UA based on model 2. Model 4 continued to adjust for BMI, and hypertension status based on model 3. Restricted cubic spline (RCS) regression was used to visualize the dose–response association of the significant association found in the logistic regression by setting six knots at 2, 4, 6, 8, 10, and 12 h. However, it needs to be emphasized that in this cross-sectional study, we are not able to determine a causal relationship between sleep duration and Hhcy. In addition, in observational studies, recall methods are the most valid and commonly used instrument to collect sleep-duration information, and it is subject to lower measuring accuracy and efficiency, and there may be measurement errors.

### Sensitivity analysis

To verify the stability of the results, the analysis was performed again on 2897 overweight or obese subjects. According to the BMI of the subjects, the subjects with BMI ≥ 30 kg/m^2^ were classified as obese, 25 kg/m^2^ ≤ BMI < 30 kg/m^2^ is overweight [21]. A multivariate logistic regression model was used to explore the relationship between sleep duration and the risk of Hhcy in the overweight population, and RCS regression was used to explore the change in sleep duration and the risk of Hhcy.

## Results

### Baseline characteristics of participants

The baseline characteristics of the study population are listed in Table 1. Table 1 presents the characteristics of the total population and is stratified by sleep duration. Comparison was made with the participants with a sleep duration of 7 h. Alcohol intake, total SFA, folic acid, vitamin B12, HDL-C, UA, TC, and BMI did not differ significantly across different sleep-duration groups (*p* > 0.05). By contrast, age, men, non-Hispanic white, current smoker, ≥ $20,000 annual household income, total energy, fat, carbohydrate, dietary fiber, MUFA, PUFA, and hypertension status varied significantly across different sleep-duration groups (*p* < 0.05).

**Table1:** Baseline characteristics in terms of quintiles of different sleep duration: NHANES, 2005–2006 (*N*=4173)

Variables	Q1 (*N* = 1,123)	Q2 (*N* = 648)	Q3 (*N* = 963)	Q4 (*N* = 1,115)	Q5 (*N* = 324)	*P* value
Age, years	48.1(17.6)	48.9(17.7)	49.1(17.8)	51.9(19.6)	53.9(21.9)	< 0.001
Men, %	581(51.7)	344(53.1)	497(51.6)	586(52.6)	142(43.8)	0.066
Non-Hispanic White, %	626(55.7)	254(39.2)	432(44.9)	620(55.6)	186(57.4)	< 0.001
Current smoker, %	226(20.1)	201(31.0)	243(25.2)	207(18.6)	79(24.4)	< 0.001
≥ $20,000 annual household income%	930(82.8)	479(73.9)	767(79.6)	867(77.8)	237(73.1)	< 0.001
Alcohol intake, drinks/day	2.8(2.7)	4.5(39.2)	2.9(3.1)	4.5(42.3)	2.7(2.6)	0.522
Total energy, kcal/day	2,112.2(821.9)	2,091.9(934)	2,077(843.6)	2,062.1(817.6)	1,909.2(772.9)	0.002
Total protein, g/day	83.3(35.9)	80.5(39.0)	80.9(37.5)	81.9(34.9)	75(35.6)	0.016
Total fat, g/day	78.7(37.3)	79(42.3)	79.3(41)	77.4(36.7)	71.5(36.1)	0.030
Total carbohydrate, g/day	256.5(106.6)	256.1(117.9)	250.9(103.7)	252.1(106.5)	234.7(101.5)	0.010
Total dietary fiber, g/day	16.3(8.4)	14.8(8.6)	15.4(8.0)	16.5(8.4)	14.2(7.1)	0.027
Total SFA, g/day	25.8(13.5)	25.9(14.6)	26.0(14.7)	25.9(13.8)	24.4(14.1)	0.475
Total MUFA, g/day	29.2(14.9)	29.2(16.4)	29.2(15.8)	28.5(14.2)	25.9(13.9)	0.009
Total PUFA, g/day	16.9(9.0)	17.1(10.6)	17.2(10.1)	16.3(8.7)	14.9(8.3)	0.007
Total folic acid, mg/day	415.2(267.5)	381.8(266.6)	386.9(241.2)	411.7(262.8)	355.6 (205.8)	0.094
Total vitamin B12, mg/day	5.8 (8.8)	5.7(11.5)	5.4(9.0)	5.7(7.8)	4.8(4.2)	0.240
HDL-C, mg/dL	54.2(16.4)	53.5(16.9)	53.9(15.6)	53.9(16.2)	54.8(16.5)	0.880
TC, mg/dL	198(43.6)	197.1(45.7)	195.2(38.6)	197.2(41.1)	194.9(41.3)	0.282
UA, mg/dL	5.4(1.4)	5.5(1.4)	5.5(1.5)	5.4(1.3)	5.4(1.5)	0.645
Hypertension, %	187(16.7)	139(21.5)	190(19.7)	226(20.3)	79(24.4)	0.014
BMI, kg/m^2^	28.2(5.8)	30.1(7.4)	29.1(6.7)	28.3(6.4)	28.6(9)	0.750

Continuous variables are presented as the means (*SD*). Categorical variables are presented as the numerical value (percentage)

*Q* Quintile, *N* Sample size, *SFA* Saturated fatty acid, *MUFA* Monounsaturated fatty acid, *PUFA* Polyunsaturated fatty acids, *HDL-C* high-density lipoprotein cholesterol, *UA* uric acid, *TC* total cholesterol, *BMI* Body mass index

### The association between sleep duration and the risk of hyperhomocysteinemia

The association of sleep duration with Hhcy risk was examined using multivariate logistic regression models, as provided in Table 2. The results suggested that compared with sleep duration (7 h), both excessive sleep (sleep duration ≥ 9 h) and lack of sleep (sleep duration ≤ 5 h) increase the risk of Hhcy after controlling for age, gender, smoking, drinking, income, race, total energy, fat, dietary fiber, carbohydrate, protein, SFA, MUFA, PUFA, folic acid, vitamin B12, HDL-C, TC, UA, BMI, and hypertension status.

**Fig. 1:**
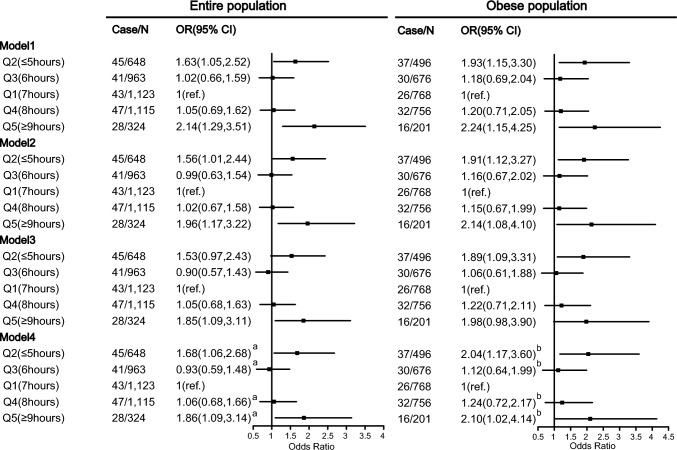
The relationship between different sleep duration and the risk of Hhcy by logistic regression models. Model 1 was adjusted age, gender, smoking, drinking, income, and race. Model 2 was further adjusted by total energy intake, total fat intake, total dietary-fiber intake, total carbohydrate intake, total protein intake, total SFA intake, total MUFA, total PUFA, total folic acid, and total vitamin B12 intake based on model 1. Model 3 was further adjusted by HDL-C, TC and UA on the basis of Model 2. Model 4a was further adjusted by BMI and hypertension based on model 3. Model 4b was further adjusted by hypertension based on model 3. *HDL-C* high-density lipoprotein cholesterol; *UA* uric acid; *TC* total cholesterol; *BMI* Body mass index; *SFA* Saturated fatty acid; *MUFA* Monounsaturated fatty acid; *PUFA* Polyunsaturated fatty acids

In the entire population, compared with the 7 h sleep-duration group (Q1), adjusted ORs of Hhcy in Q2–Q5 were 1.68 (95% CI 1.06–2.68), 0.93 (95% CI 0.59–1.48), 1.06 (95% CI 0.68–1.66), and 1.86 (95% CI 1.09–3.14), respectively. The results were shown in Fig. 1.

**Fig. 2:**
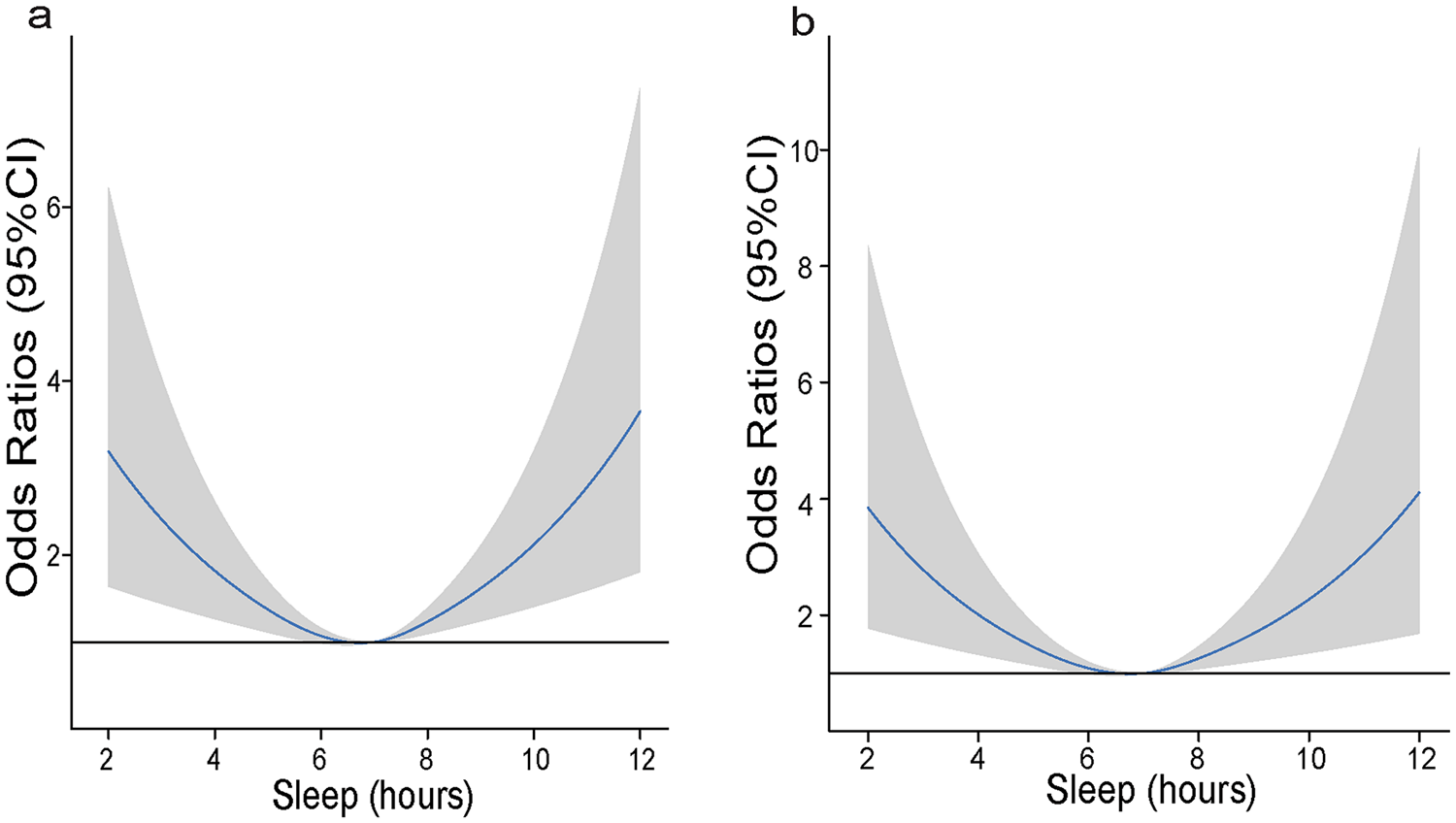
Association between different sleep duration and the risk of Hhcy in RCS in the entire population (**a**) and the overweight population (**b**). The model was adjusted for age, gender, smoking, drinking, income, race, total energy intake, total fat intake, total dietary-fiber intake, total carbohydrate intake, total protein intake, total SFA intake, total MUFA intake, total PUFA intake, total folic acid, total vitamin B12, HDL-C, TC, UA, BMI and hypertension. *HDL-C* high-density lipoprotein cholesterol; *UA* uric acid; *TC* total cholesterol; *BMI* Body mass index; *SFA* Saturated fatty acid; *MUFA* Monounsaturated fatty acid; *PUFA* Polyunsaturated fatty acids. Solid line, OR; shade, 95% CI

Because of the significant positive association of excessive or lack of sleep with the risk of Hhcy, the RCS was used to model flexibly for visualizing the above association and the risk of Hhcy was distributed in a U-shape with sleep duration, which is presented in Fig. 2a.

### Sensitivity analysis

Sensitivity analysis is an important method to verify the stability of the results and is an important part of statistical analysis in epidemiologic studies. Being overweight or obese is known to be a risk factor for Hhcy. Therefore, we performed a sensitivity analysis comparing the results of the overweight population with the entire population. The population with a sleep duration of 7 h was used as the reference standard. In the overweight population, ORs of Hhcy in Q2–Q5 were 2.04 (95% CI 1.17–3.60), 1.12 (95% CI 0.64–1.99), 1.24 (95% CI 0.72–2.17), and 2.10 (95% CI 1.02–4.14), respectively. The RCS curve result is shown in Fig. 2b.

## Discussion

To our knowledge, this is the first study to show an association between sleep duration and the incidence of Hhcy using a nationally representative sample in the United States. These results showed that excessive sleep or sleep deprivation was associated with Hhcy risk, and the associations were U-shaped between sleep duration and the risk of Hhcy. This highlighted the importance of a proper amount of sleep duration for the prevention of Hhcy.

Sleep is a reversible state of rest and recuperation with many critical active processes that comprises approximately one-third of human life. The American Academy of Sleep Medicine reported in 2015 that adults should sleep 7 or more hours per night regularly to promote optimal health [22]. A recent meta-analysis of prospective studies reports that 7–8 h of sleep per day results in the lowest risk of diabetes, with a 9% increased risk of diabetes for every hour of sleep lost [23]. Sleeping less than 7 h per night regularly is associated with adverse health outcomes, including weight gain and obesity, diabetes, hypertension, heart disease, stroke, depression [24], and increased risk of death [25]. Sleeping less than 7 h per night is also associated with impaired immune function, increased pain, impaired performance, increased errors, and greater risk of accidents [26]. Based on the above considerations, reduced sleep duration has been clearly associated with decreased quality of life as well as increased rates of metabolic disease, cardiovascular disease, and mortality [1, 27].

A previous study has demonstrated that a long sleep duration was associated with increased serum-Hcy levels, which were verified in our study, and amplified the genetic susceptibility to higher Hcy [28]. Dietary factors, as an important regulatory factor, play a critical role in the prevention and management of Hhcy. Previous study demonstrated that increased homocysteine levels were related to short sleep duration, and did not take dietary factors into account in the statistical analysis. In our study, we found that the association between sleep duration and the risk of Hhcy was U-shaped after adjusting for a series of dietary factors, which highlighted the importance of dietary factors in the relationship between sleep duration and Hhcy. Previous study has demonstrated that insomnia or hypersomnia is an independent risk factor for Hhcy. Our results are consistent with previous research. In addition, folic acid and vitamin B12 deficiency are closely related to the occurrence and development of Hhcy, and thromboembolic manifestations are relatively frequent in patients with intermediate/severe Hhcy related to inherited disorders and deficiencies in vitamin B12 and folic acid [29]. Therefore, during the statistical analysis, confounders adjusted in logistic regression included two important factors, total dietary folic acid and vitamin B12 intake.

The alteration of circadian patterns could be a key mechanism to explain our observations. The effects of sleep on circadian rhythmicity have already shown that sleep duration could affect circadian perturbance and influence the manifestation of metabolic disorders [30, 31]. Current evidence suggests that circadian rhythm-related metabolic disorders affect cysteine and other synthesis mechanisms in the human body. Indeed, an underlying circadian pattern may influence serum-Hcy concentrations, and previous studies have shown that serum-Hcy levels in adults are circadian, the highest levels occur during the late evening and the lowest levels occur during the morning [32]. Small heterodimer partner (SHP), an important regulator of lipid and bile acid metabolism, is a member of the liver clock and has been shown to play critical roles in metabolic homeostasis [33, 34]. Animal studies have shown that disruption of SHP in mice alters the expression timing of genes that regulate Hcy metabolism and the liver responses to ethanol and Hcy. Moreover, SHP inhibits the transcriptional activation of betaine-homocysteine S-methyltransferase and cystathionine g-lyase required for Hcy metabolism by FOXA1 [34].

The variation in Hcy levels could be influenced by various genetic and nongenetic events. We do understand that the metabolic cycle is likely to be influenced by the biologic clock of an individual and Hcy metabolism follows a circadian rhythm [35, 36]. The day and night cycles in humans have been reported to be regulated by Circadian Locomotor Output Cycle protein Kaput genes [37]. However, controlling the nongenetic events can the genetic factors of Hcy metabolism contributing to the variations in Hcy levels and do these variations have rhythmicity.

Although associations between long sleep duration and elevated inflammatory status have been proven statistically. Sleep and immunity are bidirectionally linked. Immune system activation alters sleep, and sleep affects our body’s defense system in turn [38]. Stimulation of the immune system by microbial challenges triggers an inflammatory response, which can not only induce an increase in sleep duration and intensity but also cause a disruption of sleep depending on its magnitude and time course. In the absence of an infectious challenge, sleep appears to promote inflammatory homeostasis through effects on several inflammatory mediators, such as cytokines. Nonetheless, a recent report has shown that sleeping too long or too short can affect immune status and disrupt immune defenses. Inflammation and immune response may be responsible for both cytokine production and telomeric erosion[39], and another study has highlighted that shortened leukocyte telomere length is associated with increased Hcy levels [40]. As can be seen from the above, sleep duration may play a role in regulating levels of Hcy. This notion is supported by findings that prolonged sleep deficiency (e.g., short sleep duration, sleep disturbance) can lead to chronic, systemic low-grade inflammation and is associated with various diseases that have an inflammatory component, like diabetes, atherosclerosis, Hhcy, and neurodegeneration.

We found that increasing age was a risk factor for Hhcy, consistent with previous epidemiologic findings. A large number of investigations and studies have shown that the prevalence of Hhcy caused by population aging increases with age, the body’s immunity decreases, multiple organs weaken progressively, and the number of hospitalizations and invasive procedures increase, which makes viruses and bacteria more invasive and people more susceptible to infection [38]. The induction of a hormonal constellation that supports immune functions is one likely mechanism underlying the immune-supporting effects of sleep.

To conclude, short sleep duration and oversleeping are related to a variety of diseases, including Hhcy. One of the most distinctive features of this study is we discovered the association between sleep duration and Hhcy risk by performing a large sample quantitative data analysis on the NHANES database. Meanwhile, we adjusted for confounding factors including dietary factors associated with sleep or Hcy at various points in the multivariate logistic regression models. This study still has some limitations and needs to be further improved: First, in this cross-sectional study, sleep duration was evaluated by self-reported questionnaires and serum homocysteine levels were assessed only at one time, which might lead to certain bias and could not make causal inferences. In the following research, it is necessary to further use mendelian randomization method to explore the causal relationship between sleep duration and Hhcy. Further longitudinal investigations concerning the effect of sleep duration on homocysteine alteration might help provide a better understanding of the pathogenesis of Hhcy. Second, some risk factors associated with Hhcy might not be identified in the study, which might lead to result bias. Therefore, when we investigate the causal relationship between sleep duration and Hhcy, confounding factors should be adjusted as comprehensively as possible. Third, there may be a link between chronotype and homocysteine. However, the variables provided by NHANES database were limited, and the chronotype variable was not included in the NHANES database. Therefore, the effect of chronotype on Hhcy was not considered in this study, and this variable would be fully considered in the following mechanism study.

## Conclusion

In this cross-sectional study, both short- and long-sleep durations were associated with worse outcomes for Hhcy. The association is U-shaped between sleep duration and the risk of Hhcy emphasizing the importance of maintaining adequate sleep. Appropriate sleep duration is necessary to prevent the occurrence and development of Hhcy.

## References

[1] Yamamoto R, Shinzawa M, Isaka Y, et al. sleep quality and sleep duration with CKD are associated with progression to ESKD. Clin j Am Soc Nephrol: CJASN. 2018;13(12):1825–32. 30442866 10.2215/CJN.01340118 PMC6302324

[2] Reis C, Dias S, Rodrigues AM, et al. Sleep duration, lifestyles and chronic diseases: a cross-sectional population-based study. Sleep Sci. 2018;11(4):217–30. 30746039 10.5935/1984-0063.20180036 PMC6361301

[3] Chattu VK, Manzar MD, Kumary S, et al. The global problem of insufficient sleep and its serious public health implications. Healthcare (Basel, Switzerland). 2018. 10.3390/healthcare7010001. 30577441 10.3390/healthcare7010001 PMC6473877

[4] Warsame F, Chu NM, Hong J, et al. Sleep duration and cognitive function among older adults with chronic kidney disease: results from the national health and nutrition examination survey (2011–2014). Nephrol Dial Transplant Publ Eur Dial Transplant Assoc Eur Ren Assoc. 2023;38(7):1636–44. 10.1093/ndt/gfac325 PMC10310518 36535636

[5] Owens OJE, R L. Chronic obstructive pulmonary disease and breathing during sleep. a strain in the neck. Am J Res Critical Care Med. 2020;201:395–6. 10.1164/rccm.201911-2174ED PMC7049932 31810375

[6] Depner CM, Wright SER, K P, Jr. Metabolic consequences of sleep and circadian disorders. Curr Diabt Reports. 2014;14:507. 10.1007/s11892-014-0507-z PMC4308960 24816752

[7] Hirshkowitz M, Whiton K, Albert SM, et al. National sleep foundation’s updated sleep duration recommendations: final report. Sleep Health. 2015;1(4):233–43. 29073398 10.1016/j.sleh.2015.10.004

[8] Zaric BL, Obradovic M, Bajic V, et al. Homocysteine and hyperhomocysteinaemia. Curr Med Chem. 2019;26(16):2948–61. 29532755 10.2174/0929867325666180313105949

[9] Gambhir DS. Homocysteinemia and risk for cardiovascular disease. Indian Heart J. 2000;52(7 Suppl):S2-4. 11339436

[10] Rajagopalan P, Hua X, Toga AW, et al. Homocysteine effects on brain volumes mapped in 732 elderly individuals. NeuroReport. 2011;22(8):391–5. 21512418 10.1097/WNR.0b013e328346bf85 PMC3192851

[11] Trześniowska-Drukała B, Kalinowska S, Safranow K, et al. Evaluation of hyperhomocysteinemia prevalence and its influence on the selected cognitive functions in patients with schizophrenia. Prog Neuro-Psychopharmacolbiol Psych. 2019;95:109679. 10.1016/j.pnpbp.2019.109679 31254573

[12] Mu L, Lin Y, Huang X, et al. Sex differences in the prevalence and clinical correlates of hyperhomocysteinemia in patients with bipolar disorder. Hum Psychopharmacol. 2020;35(2): e2724. 32052509 10.1002/hup.2724

[13] Paul B, Saradalekshmi KR, Alex AM, et al. Circadian rhythm of homocysteine is hCLOCK genotype dependent. Mol Biol Rep. 2014;41(6):3597–602. 24510388 10.1007/s11033-014-3223-5

[14] Xie Z, Chen F, Li WA, et al. A review of sleep disorders and melatonin. Neurol Res. 2017;39(6):559–65. 28460563 10.1080/01616412.2017.1315864

[15] Champier J, Claustrat F, Nazaret N, et al. Folate depletion changes gene expression of fatty acid metabolism, DNA synthesis, and circadian cycle in male mice. Nutr Res. 2012. 10.1016/j.nutres.2011.12.012. 22348461 10.1016/j.nutres.2011.12.012

[16] Vasey C, McBride J, Penta K. Circadian rhythm dysregulation and restoration: the role of melatonin. Nutrients. 2021. 10.3390/nu13103480. 34684482 10.3390/nu13103480 PMC8538349

[17] Beydoun MA, Gamaldo AA, Canas JA, et al. Serum nutritional biomarkers and their associations with sleep among US adults in recent national surveys. PLoS ONE. 2014;9(8): e103490. 25137304 10.1371/journal.pone.0103490 PMC4138077

[18] Li K, Zhang J, Qin Y, et al. Association between serum homocysteine level and obstructive sleep apnea: a meta-analysis. BioMed Res Int. 2017. 10.1155/2017/7234528. 28831396 10.1155/2017/7234528 PMC5555021

[19] Shan Z, Rehm CD, Rogers G, et al. Trends in dietary carbohydrate, protein, and fat intake and diet quality among US adults, 1999–2016. JAMA. 2019;322(12):1178–87. 31550032 10.1001/jama.2019.13771 PMC6763999

[20] Morris MS, Bostom AG, Jacques PF, et al. Hyperhomocysteinemia and hypercholesterolemia associated with hypothyroidism in the third US national health and nutrition examination survey. Atherosclerosis. 2001;155(1):195–200. 11223442 10.1016/s0021-9150(00)00537-2

[21] Bult MJ, van Dalen T, Muller AF. Surgical treatment of obesity. Eur J Endocrinol. 2008;158(2):135–45. 18230819 10.1530/EJE-07-0145

[22] Watson NF, Badr MS, Belenky G, et al. Recommended amount of sleep for a healthy adult: a joint consensus statement of the American Academy of Sleep Medicine and Sleep Research Society. Sleep. 2015;38(6):843–4. 26039963 10.5665/sleep.4716 PMC4434546

[23] Shan Z, Ma H, Xie M, et al. Sleep duration and risk of type 2 diabetes: a meta-analysis of prospective studies. Diabetes Care. 2015;38(3):529–37. 25715415 10.2337/dc14-2073

[24] Moradi F, Lotfi K, Armin M, et al. The association between serum homocysteine and depression: a systematic review and meta-analysis of observational studies. Eur J Clin Invest. 2021;51(5): e13486. 33423269 10.1111/eci.13486

[25] Cappuccio FP, D’Elia L, Strazzullo P, et al. Sleep duration and all-cause mortality: a systematic review and meta-analysis of prospective studies. Sleep. 2010;33(5):585–92. 20469800 10.1093/sleep/33.5.585 PMC2864873

[26] Bollinger T, Bollinger A, Oster H, et al. Sleep, immunity, and circadian clocks: a mechanistic model. Gerontology. 2010;56(6):574–80. 20130392 10.1159/000281827

[27] Åkerstedt T, Ghilotti F, Grotta A, et al. Sleep duration and mortality-does weekend sleep matter? J Sleep Res. 2019;28(1): e12712. 29790200 10.1111/jsr.12712 PMC7003477

[28] Mo T, Wang Y, Gao H, et al. Sleep duration, midday napping, and serum homocysteine levels: a gene-environment interaction study. Nutrients. 2023. 10.3390/nu15010210. 36615867 10.3390/nu15010210 PMC9823917

[29] Guéant JL, Guéant-Rodriguez RM, Oussalah A, et al. Hyperhomocysteinemia in cardiovascular diseases: revisiting observational studies and clinical trials. Thromb Haemost. 2023;123(3):270–82. 36170884 10.1055/a-1952-1946

[30] Matenchuk BA, Kozyrskyj MPJ, A L. Sleep, circadian rhythm, and gut microbiota. Sleep med rev. 2020;53:101340. 32668369 10.1016/j.smrv.2020.101340

[31] Borbély AA, Daan S, Wirz-Justice A, et al. The two-process model of sleep regulation: a reappraisal. J Sleep Res. 2016;25(2):131–43. 26762182 10.1111/jsr.12371

[32] Bremner WF, Holmes EW, Kanabrocki EL, et al. Circadian rhythm of serum total homocysteine in men. Am j cardiol. 2000;86:1153–6. 11074221 10.1016/s0002-9149(00)01181-4

[33] Marczak MM, Yan B. Circadian rhythmicity: A functional connection between differentiated embryonic chondrocyte-1 (DEC1) and small heterodimer partner (SHP). Arch biochem biophy. 2017;631:11–8. 10.1016/j.abb.2017.08.004 PMC5599142 28797635

[34] Tsuchiya H, da Costa KA, Lee S, et al. Interactions between nuclear receptor SHP and FOXA1 maintain oscillatory homocysteine homeostasis in mice. Gastroenterology. 2015;148(5):1012-1023.e1014. 25701738 10.1053/j.gastro.2015.01.045 PMC4409521

[35] Martins PJ, Galdieri LC, Souza FG, et al. Physiological variation in plasma total homocysteine concentrations in rats. Life Sci. 2005;76(22):2621–9. 15769485 10.1016/j.lfs.2004.12.011

[36] Lavie L, Lavie P. Daily rhythms in plasma levels of homocysteine. J Circadian Rhythms. 2004;2(1):5. 15347422 10.1186/1740-3391-2-5 PMC520826

[37] Katzenberg D, Young T, Finn L, et al. A clock polymorphism associated with human diurnal preference. Sleep. 1998;21(6):569–76. 9779516 10.1093/sleep/21.6.569

[38] Besedovsky L, Lange T, Haack M. The sleep-immune crosstalk in health and disease. Physiol rev. 2019;99:1325–80. 30920354 10.1152/physrev.00010.2018 PMC6689741

[39] Wong JY, De Vivo I, Lin X, et al. The relationship between inflammatory biomarkers and telomere length in an occupational prospective cohort study. PLoS ONE. 2014;9(1): e87348. 24475279 10.1371/journal.pone.0087348 PMC3903646

[40] Zhang D, Wen X, Wu W, et al. Homocysteine-related hTERT DNA demethylation contributes to shortened leukocyte telomere length in atherosclerosis. Atherosclerosis. 2013;231(1):173–9. 24125430 10.1016/j.atherosclerosis.2013.08.029

